# Microscopical Evaluation of the Effects of High-Pressure Processing on Milk Casein Micelles

**DOI:** 10.3390/molecules27217179

**Published:** 2022-10-24

**Authors:** Sergio O. Serna-Hernandez, Zamantha Escobedo-Avellaneda, Rebeca García-García, Magdalena de Jesús Rostro-Alanis, Jorge Welti-Chanes

**Affiliations:** Escuela de Ingeniería y Ciencias, Tecnologico de Monterrey, Eugenio Garza Sada 2501, Monterrey 64700, NL, Mexico

**Keywords:** casein micelle, scanning electron microscopy, high-pressure processing, milk

## Abstract

The effect of different high-pressure processing (HPP) treatments on casein micelles was analyzed through scanning electron microscopy (SEM) and a particle size distribution analysis. Raw whole and skim milk samples were subjected to HPP treatments at 400, 500 and 600 MPa for Come-Up Times (CUT) up to 15 min at ambient temperature. Three different phenomena were observed in the casein micelles: fragmentation, alterations to shape and agglomeration. The particle size distribution analysis determined that, as pressure and time treatment increased, the three phenomena intensified. First, the size of the casein micelles began to decrease as their fragmentation occurred. Subsequently, the casein micelles lost roundness, and their shape deformed. Finally, in the most intense treatments (higher pressures and/or longer times), the micelles fragments began to agglomerate, which resulted in an increase in their average diameter. Homogenization and defatting had no significant effect on the casein micelles; however, the presence of fat in whole milk samples was bioprotective, as the effects of the three phenomena appeared faster in treated skim milk samples. Through this study, it was concluded that the size and structure of casein micelles are greatly altered during high-pressure treatment. These results provide information that broadens the understanding of the changes induced on casein micelles by high-pressure treatments at room temperature.

## 1. Introduction

High-Pressure Processing (HPP) is a non-thermal food preservation technology that is capable of inactivating pathogenic and spoilage microorganisms whilst having a minimal impact on the sensory and nutritional characteristics of foods [1,2,3]. The application of HPP in milk has been considered a viable alternative to traditional thermal pasteurization processes and achieves a minimum of a 5 log_10_ microbial reduction and 20-day shelf life upon refrigeration [1,2,3,4,5,6,7,8]. It has been reported that treatments of raw milk at pressure intensities of 600 MPa for a few minutes are effective in the inactivation of pathogenic microorganisms, with results equivalent to those obtained with traditional thermal pasteurization processes; thus, HPP also extends the shelf life and guarantees the safety of milk [2,3,4]. As previously reported by Serna-Hernandez et al. [3], HPP also has an effect on milk proteins, specifically on the structure of casein micelles, and milk fat globules.

Casein is the most abundant protein fraction in milk, encompassing approximately 80% of the total protein content. There are four different types of casein molecules: α_s1_, α_s2_, β and κ caseins [9]. In milk, casein alongside calcium phosphate can bind and form aggregates that are known as casein micelles, which have an average size, in a native state, of 150 to 200 nm and are highly hydrated [9,10]. The proportion of the different types of casein molecules in the micelles can vary: κ caseins are typically found on micelles’ surface or coating, β caseins are found on their interior, and both forms of α_s_ caseins can be expected to be present along their whole structure [9]. The properties of casein micelles provide many of the technologically important characteristics of milk such as the white color, stability to heat or ethanol, and coagulation by rennet [11,12]. The disruption of casein micelles causes different physicochemical changes in milk, as its structure is altered through processing, the functionality of milk proteins changes, and parameters such as pH, acidity, color, turbidity and particle size distribution could be altered [11]. In addition, milk proteins represent the basic structure of dairy foods, which owe their sensory and textural attributes primarily to the formation of protein networks by heat, pH, enzymatic modification or a combination of these factors [11].

An important detail to highlight regarding casein micelles is that, up to this date, the exact and accurate structure, nature and composition of these aggregates are highly debated [9,10]. The direct observation of the structures of nanoparticles and nanocolloids in liquid samples has contributed to the analysis of their functions and mechanisms [12,13]. Microscopy techniques, such as scanning electron microscopy (SEM), can be used to visualize micelles and observe changes caused by HPP by providing an analysis and the interpretation of the resulting micrographs [9,10,11,12,13,14]. The aim of this study was to evaluate the effects of different HPP treatments on milk casein micelles utilizing a scanning electron microscope (SEM) technique and advance the understanding of how the observed changes could affect the quality and stability of milk treated by HHP.

## 2. Results

Table 1 displays the pH, acidity, protein content, fat content and total solid content of the whole and skim milk samples which were analyzed as the milk samples were received. The values obtained are within the quality ranges according to NOM-155-SCFI-2012 [15]. This confirmed the quality of the milk used for the experiments.

### Effect of the HPP Treatments on the Diameter of the Casein Micelles 

**Figure 1:**
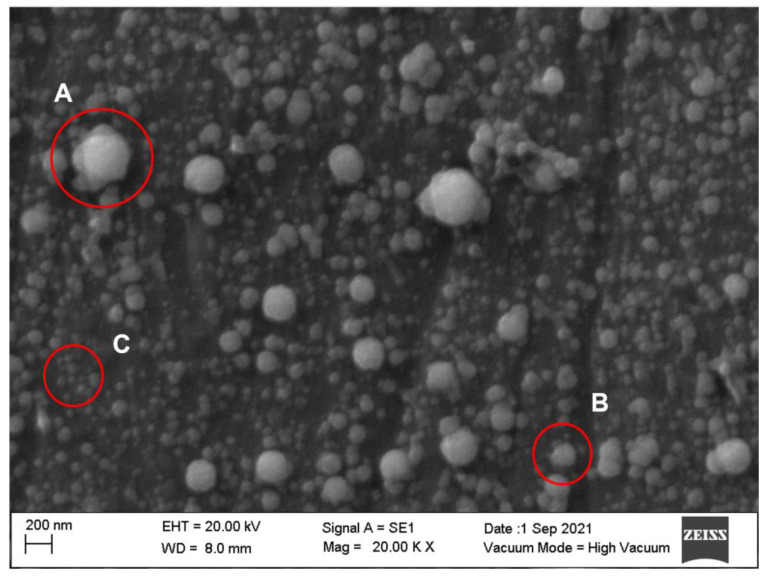
Scanning electron microscope micrograph of milk casein micelles treated at 600 MPa for 2 min. Examples of agglomeration (**A**), alteration to the shape (**B**) and micelle fragments (**C**).

To facilitate the visualization and interpretation of the obtained SEM micrographs, Figure 1 was created. Figure 1 contains an example of a milk sample subjected to 600 MPa, which displays all three of the proposed and observed phenomena in this study. Agglomeration (A), which can be described as casein micelles of different sizes that are stuck together, alterations to the micelles’ shape (B), characterized by the loss of roundness, and fragmentation (C), i.e., the breaking of the micelles into small participles due to the high-pressure process.

**Figure 2:**
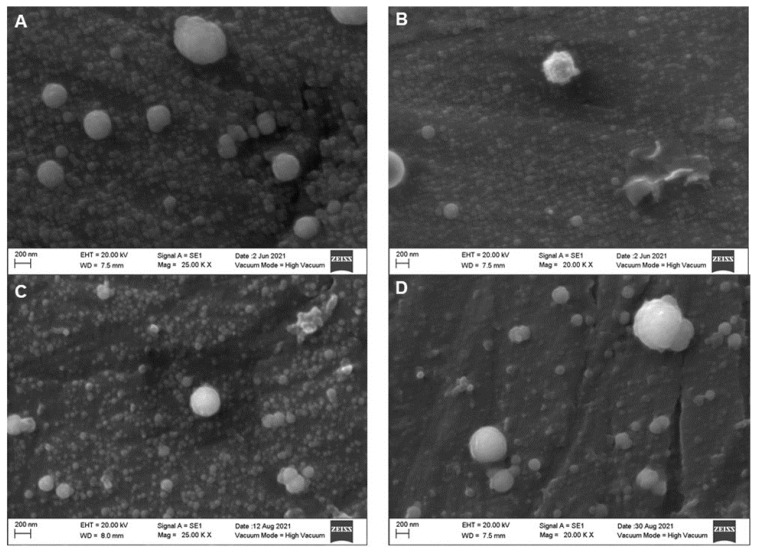
Scanning electron microscopy micrographs of an untreated milk sample. Whole, unhomogenized, raw milk (**A**); whole, homogenized raw milk (**B**); skim, unhomogenized raw milk (**C**); skim, homogenized raw milk (**D**).

**Figure 3:**
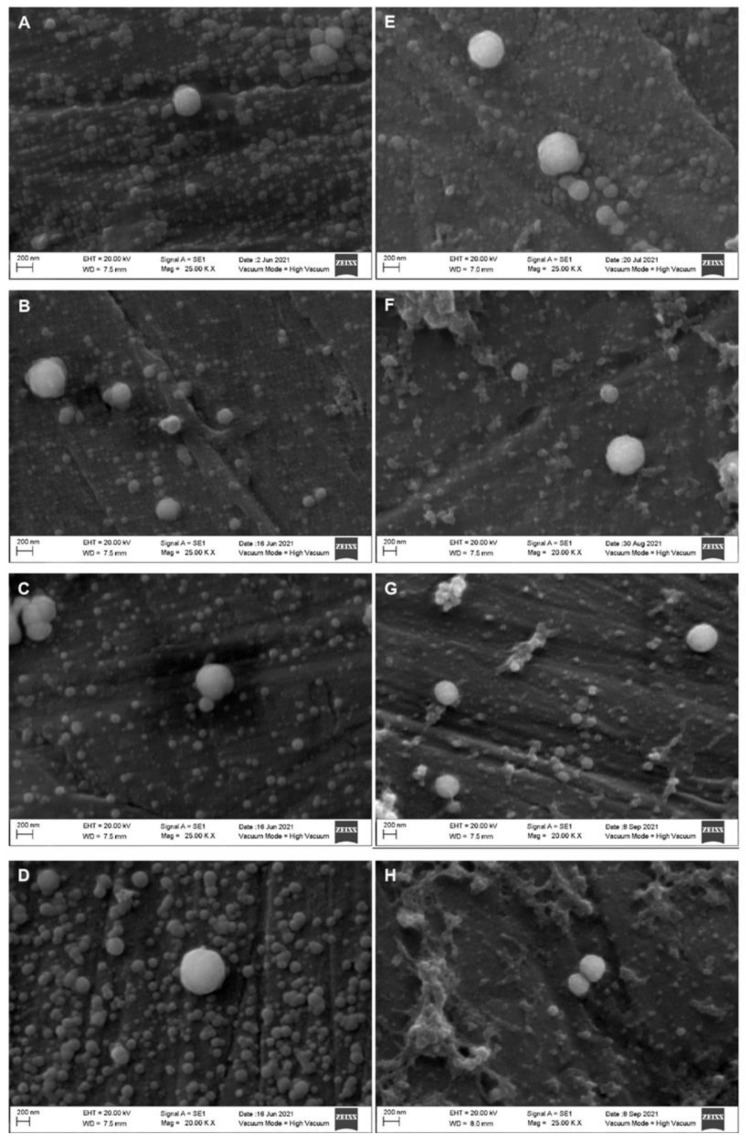
Scanning electron microscopy micrographs of homogenized whole (left column) and skim (right column) milk samples treated at 400 MPa. Come-Up Time (**A**,**E**); 2 min (**B**,**F**); 5 min (**C**,**G**); 10 min (**D**,**H**).

**Figure 4:**
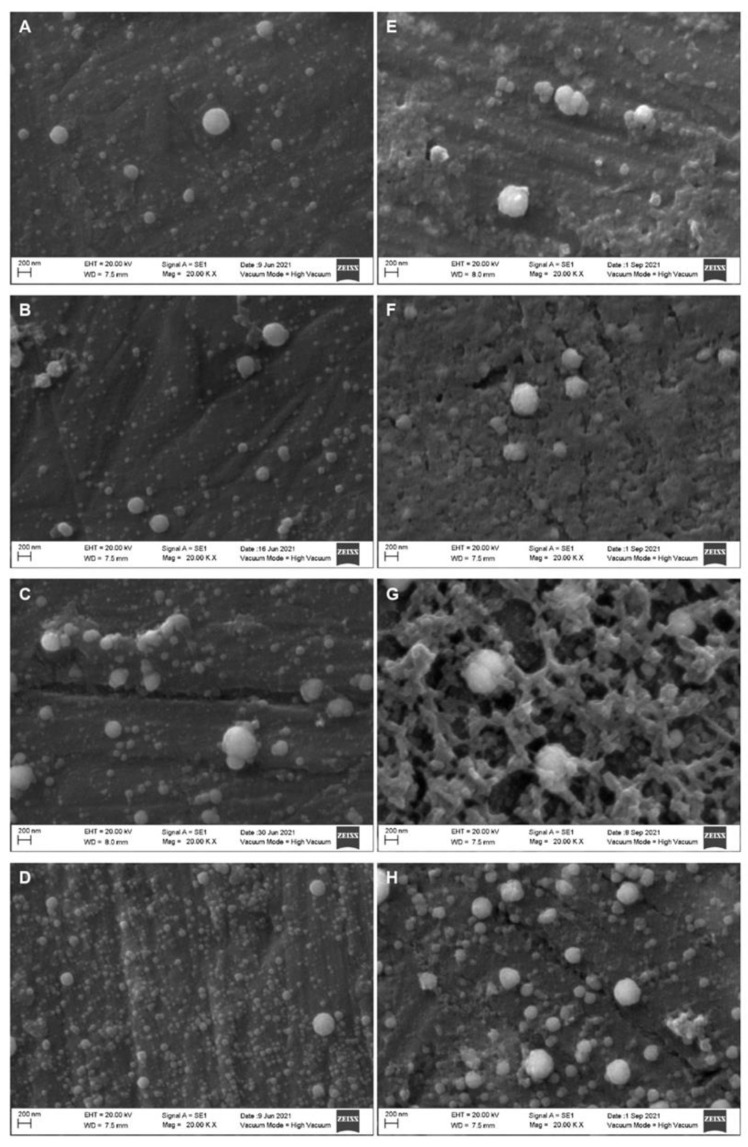
Scanning electron microscopy micrographs of homogenized whole (left column) and skim (right column) milk samples treated at 500 MPa. Come-Up Time (**A**,**E**); 2 min (**B**,**F**); 5 min (**C**,**G**); 10 min (**D**,**H**).

**Figure 5:**
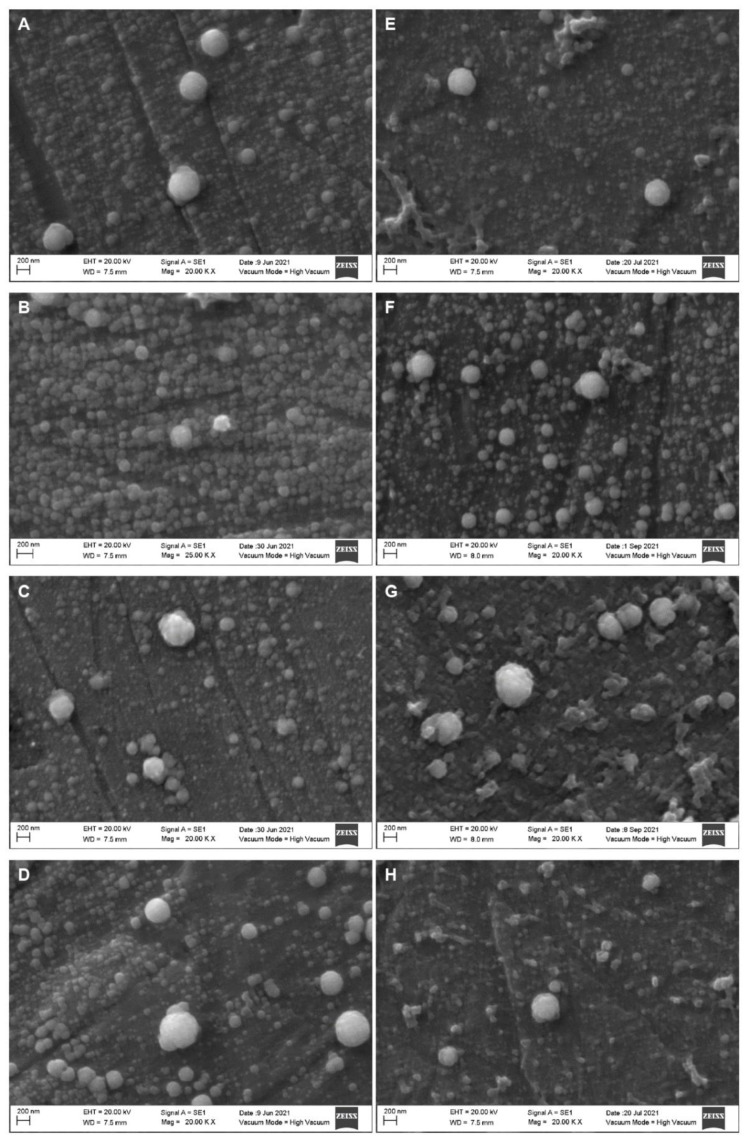
Scanning electron microscopy micrographs of homogenized whole (left column) and skim (right column) milk samples treated at 600 MPa. Come-Up Time (**A**,**E**); 2 min (**B**,**F**); 5 min (**C**,**G**); 10 min (**D**,**H**).

Figure 2 displays the SEM micrographs of four different types of raw milk. As seen, the size of the casein micelles varied; in the background, smaller fragmented particles can be distinguished, whereas bigger, rounder, and complete ones are observed in the foreground. SEM micrographs of 400-, 500- and 600 MPa-treated milk samples are displayed in Figure 3, Figure 4 and Figure 5, respectively. When compared to the treated samples, the raw milk samples in Figure 2 displayed individual bigger micelles and more particle fragments in the background. In general, the size, shape and accommodation of milk casein micelles is affected by high-pressure processing. The intensity of a treatment refers to its pressure levels and duration. As the intensity of the high-pressure treatment increases, the size of the casein micelle decreases due to fragmentation, the shape of the particles is altered, as roundness tends to be lost, and in the most intense treatments, the fragmented casein micelles tend to agglomerate and generate joint particles. In general, the CUT during 5 min of treatment yielded the smaller particle size distribution of the casein micelles, as fragmentation was promoted. Subsequent treatments for 8 and 10 min led to particles with bigger sizes, attributed to the agglomeration.

Figure 3 displays whole and skim milk samples treated at 400 MPa. It can be observed that the as the treatment time increased, the frequency and size of the casein micelles decreased, and the frequency of the smaller fragments in the background increased. Additionally, with the longer treatment times, smaller casein micelle fragments were observed in the background, and the incidence of agglomeration decreased. The micrographs of whole and skim milk samples treated at 500 MPa are displayed in Figure 4. When compared to Figure 3, the size of the casein micelles was smaller, and the background became saturated with fragments of smaller casein micelles more quickly. Additionally, the agglomeration and shape alteration phenomena predominated, especially during the longer treatment times, such as the 10 min treatment. Finally, Figure 5 displays the micrographs of the whole and skim milk samples subjected to the 600 MPa treatment. For these samples, a severe casein micelle fragmentation was observed at the CUT. Moreover, agglomeration of casein micelles could also be appreciated in all four treatments, and alterations to the micelles’ shape were observed starting from the 2 min treatment.

The casein micelles’ sizes in the treated whole milk samples and the skimmed milk samples are reported in Table 2. The micelles’ mean diameters in the whole milk samples were statistically different (*p* < 0.05); overall, the untreated samples presented casein micelles with similar mean diameters. Additionally, the three different CUT treatments presented a similar behavior as similar mean diameters were observed. The homogenized and unhomogenized raw milk samples had an average casein micelle diameter of 391 and 370 nm, respectively. All three CUT treatments also had statistically similar mean diameters, i.e., 319, 273 and 237 nm at 400, 500 and 600 MPa. These values were similar for both of the untreated samples. Conversely, all 400, 500 and 600 MPa treatments, longer than the CUT, led to statistically smaller mean diameters when compared to the diameters of the untreated samples. Average diameters of 159, 198 and 166 nm were obtained with treatments at 400, 500 and 600 MPa for 5 min treatments, respectively. Additionally, slightly higher mean values were observed for the longer treatments, i.e., 208 nm at 400 MPa, 15 min, 284 and 239 nm at 500 and 600 MPa, 10 min, respectively. These results indicate micelle agglomeration, which was more important in the most intense pressure treatments. To complement these data, the average diameter size of the untreated samples was used as a baseline to calculate the reduction percentage in size considering the smallest mean diameters observed at each pressure intensity. A 57% reduction in size was observed at 400 MPa for 5 min, a 49% size reduction at 500 MPa for 2 min, and a 64% size reduction at 600 MPa for 2 min.

The results for the high pressure-treated skimmed milk samples (Table 1) were statistically different (*p* < 0.05). A similar pattern was observed, as the control samples presented statistically similar mean diameters when compared to the three CUT treatments. The average mean diameter for the control samples was 370 nm, and the CUT treatments yielded 233, 322 and 238 nm for 400, 500 and 600 MPa, respectively. The longer treatments also led to an increase in the mean diameters, i.e., 588 and 625 nm for 400 MPa, 15 min and 500 MPa, 8 min, respectively. Similarly, the reduction percentage in size when compared to the control sample was calculated. The 400 MPa, 5 min treatment led to a 29% reduction, the 500 MPa, 10 min to a 15% reduction, and the 600 MPa, 10 min, to the most considerable reduction of 36%.

**Figure 6:**
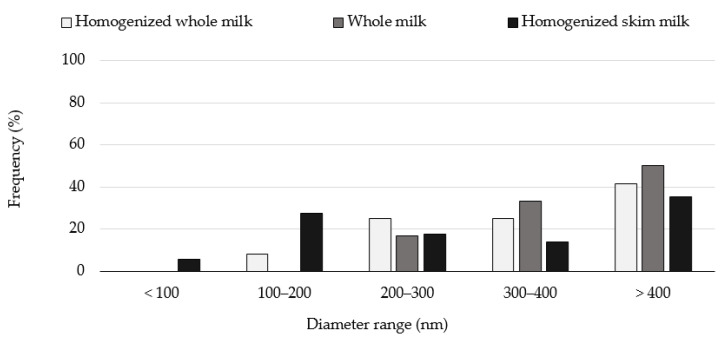
Casein micelle diameter distribution in untreated whole and skimmed milk samples. The measurements were recorded from the micrographs using the scanning electron microscopy equipment software.

Furthermore, the elevated standard deviations did not allow to observe significant statistical differences, and a distribution size analysis was conducted. Histograms were elaborated depicting the diameter range of the casein micelles and the observed frequency for each different sample group. Figure 6 displays the histograms for the untreated milk whole and skim samples. It can be observed that in all different untreated milk samples, the casein micelles’ size diameter tended to be bigger than 400 nm. This value was determined for up to 50% of the measured micelles in the untreated whole milk samples, about 41% in the homogenized milk samples and approximately 35% in the homogenized skim milk samples. In addition, as the diameter range decreased, the observed frequency decreased as well. As a point of comparison, it has been reported that the average casein micelle diameter in any milk type ranges from 100 to 300 nm [9].

**Figure 7:**
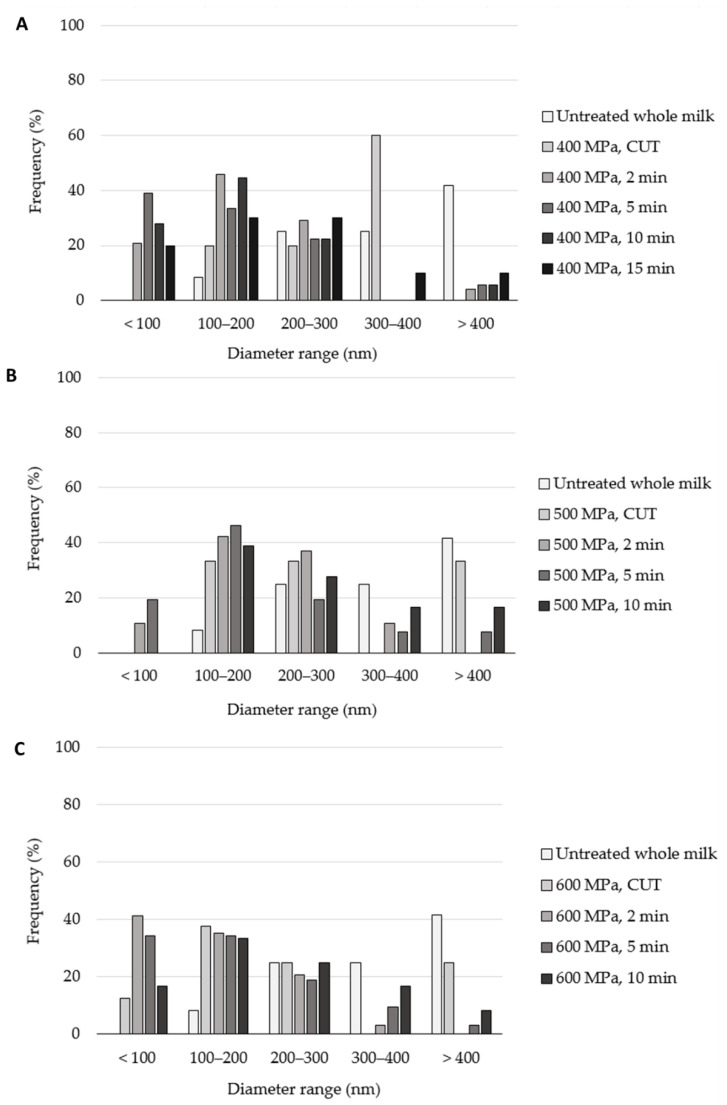
Casein micelle diameter distribution in high-pressure-treated homogenized whole milk samples at 400 (**A**), 500 (**B**) and 600 MPa (**C**). The measurements were recorded from the micrographs by the scanning electron microscopy equipment software.

Figure 7 presents the results of skim milk samples treated at 400, 500 and 600 MPa. Figure 7A depicts the results for whole milk samples treated at 400 MPa. The CUT treatment caused casein micelle fragmentation (see Figure 5F), as no micelles with a diameter of more than 400 nm were observed, and the frequency of micelles with diameters of other sizes increased when compared to the untreated samples. For example, the frequency of micelles with a diameter in the range of 300 to 400 nm for the CUT treatment was 60%, whereas for the untreated sample, it was approximately 25%. This pattern was also observed for the more intense treatment, as the frequency of the casein micelles with the smaller diameter increased as the treatment time increased. However, an additional phenomenon was observed for the longer treatments. In fact, for the 2, 5, 10 and 15 min treatments, casein micelles with sizes of 400 nm or more started to reappear, which was attributed to their agglomeration. The frequency of these micelles increased as the treatment time increased, reaching up to 10% in the 15 min treatment. The same patterns are observed in Figure 7B,C, which presents the results for whole milk treated at 500 and 600 MPa, respectively. In Figure 7B, as the treatment time increased, the frequency of the smaller diameter ranges tended to increase. However, for the 500 MPa, 10 min treatment, the opposite was seen as increased frequency for the 200 to 300, 300 to 400 and 400 nm or more diameter ranges was observed, which also was attributed to casein micelle agglomeration. In Figure 7C, both fragmentation and agglomeration can be more easily observed. The 600 MPa, CUT sample results yielded increased frequencies for the smaller diameter ranges when compared to the untreated counterpart. This behavior was exacerbated in the case of the treatment at 600 MPa, 2 min, as a frequency higher than 40% was measured for micelles with a diameter below 100 nm. For the 600 MPa 5 and 10 min samples, the agglomeration phenomenon was clearly observed when evaluating the 300 to 400 and the larger than 400 nm range frequencies, as these tended to increase alongside the treatment time.

**Figure 8:**
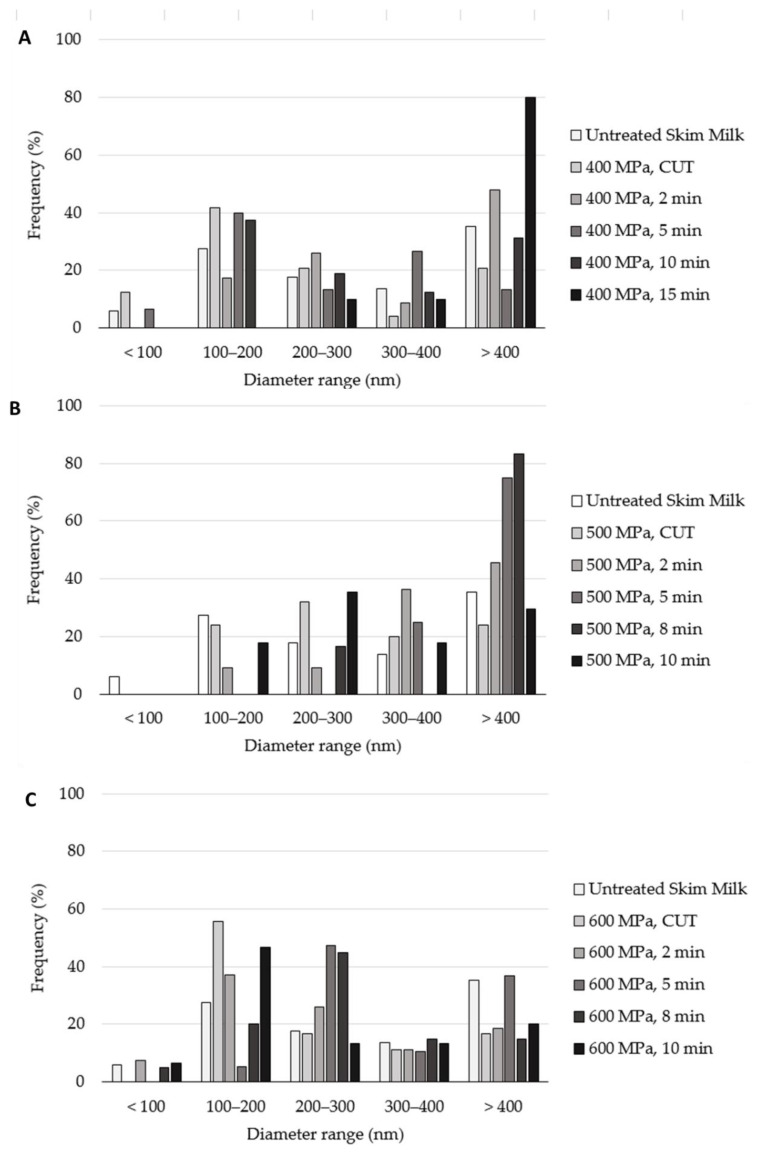
Casein micelle diameter distribution in 400 (**A**), 500 (**B**) and 600 MPa (**C**) high-pressure-treated homogenized, skim milk samples. The measurements were recorded from the micrographs by the scanning electron microscopy equipment software.

Figure 8 presents the results of skim milk samples treated at 400, 500 and 600 MPa. In Figure 8A, the 400 MPa CUT, 2 min treatment yielded an increased frequency of the smaller diameter ranges, due to casein micelle fragmentation. However, when comparing the 5, 10 and 15 min treatments, it can be seen that the frequency steadily increased for the larger diameter ranges because of agglomeration; for example, the 400 MPa, 15 min sample had an observed frequency of 80% for casein micelles bigger than 400 nm. The same was also observed for the 500 MPa-treated samples, as shown in Figure 8B. However, the sample that yielded the largest frequency of casein micelles larger than 400 nm was the 8 min treatment, not the longer 10 min treatment. Figure 8C, which displays the results regarding the 600 MPa skimmed milk samples, shows the highest frequency of micelles with a diameter bigger than 400 nm for the 600 MPa, 5 min samples. The longer treatments presented higher frequencies of the smaller diameter ranges.

Overall, after observing and analyzing the histograms depicting different diameter ranges and the observed frequency for all different milk samples, it was possible to detect that the shorter high-pressure treatment started to generate casein micelle fragmentation, and as the treatment time started to increase, the fragmentation process was promoted up to a certain point. Once this point was reached, the opposite behavior was observed, and the agglomeration of the casein micelles caused an increase in their diameter. Additionally, the time necessary for the micelles to start agglomerating appeared to decrease as the treatment pressure increased.

Moreover, the presence of fat, or lack thereof, in milk samples appeared to also affect the behavior of the micelles during high-pressure processing. From the obtained results, the presence of fat generates a source of protection for the casein micelles, as greater mean diameters were obtained in whole milk samples. This can be observed when comparing Figure 7 and Figure 8; for example, as seen in Figure 8C, the 600 MPa, CUT sample presented the most frequent casein micelle sizes in the 100 to 200 nm range; however, the 600 MPa, 5 min treatment had the most frequently observed ranges of 200 nm or more, meaning that agglomeration was observed due to the intense conditions. This behavior is proposed because in all skim milk samples, agglomeration was observed faster at the shorter treatment time, as it was previously discussed. For the skim milk samples, fragmentation was first observed, quickly followed by casein micelle agglomeration, and in the most intense and long treatments, such as the 600 MPa 8 and 10 min treatments, fragmentation was observed again. Similar results were reported by Yang et al. [16], who stated that skim milk is more susceptible to HPP than whole milk and proposed milk fat as a baroprotective component for casein micelles.

It has been reported that, during HPP, casein micelles undergo changes in their size, composition, and hydration. During pressurization, water is compressed, which generates a disruption in the hydrophobic bonding of the casein micelle components causing the micellar calcium phosphate to be solubilized [9,13,17,18,19,20,21]. More specifically, as the water molecules are compacted and compressed, they penetrate and hydrate the casein micelle molecules, which causes a dissociation of ion pairs followed by the release and solubilization of calcium phosphate [18,19,21]. These changes caused during HPP are attributed to size alterations in casein micelles. Other factors that influence the size and shape of these molecules is the presence of denatured whey proteins, which also originate from the HPP, as they can bind to the surface of the micelles [9]. Conversely, the specific mechanisms that allow casein micelle agglomeration have not been determined. As previously discussed, the most intense pressure treatments promote micellar fragmentation, which releases calcium phosphate and causes the depletion of κ-and β-casein. It has been speculated that these two mechanisms are responsible for the agglomeration that occurs in the most intense of high-pressure treatments. It is thought that, as more micellar calcium phosphate is released from the fragment particles, it also becomes more available in the medium to interact with the micelles, promoting their agglomeration. Additionally, the presence of κ-casein on the surface of casein micelles makes these particles stable against aggregation; HPP disrupts the surface and depletes this protein, which may also be responsible for the agglomeration of these particles [9].

A study in which reconstituted micellar casein concentrates and milk protein concentrations were treated at 150 to 450 MPa for 15 min reported similar results [11]. Through the interpretation of the SEM micrographs, these authors reported that the samples were significantly altered by the high-pressure treatment, and a loss of shape and a decrease in size were reported. Casein micelles with spherical shapes and diameter in the range of hundreds of nanometers changed their shapes and acquired significantly smaller diameter sizes. In addition, the authors explained the presence of a network of micellar substructures with a size in the range of 20 nm in the background of the treated samples. Additionally, Huppertz et al. [22] reported that subjecting milk to 250 MPa yielded a 25% increase in casein micelle size, whereas a treatment at 300 MPa irreversibly caused a 50% reduction in size. Hemar et al. [14] evaluated the effects of HPP in reformed casein micelles and reported an average particle size of 85 nm for samples subjected to 300, 400 and 500 MPa, which is a size reduction equivalent to 57%.

## 3. Materials and Methods

### 3.1. Milk Samples

Homogenized and unhomogenized, raw whole and skimmed milk samples were obtained directly from Grupo Alpura, Ciudad de México, México. The samples were shipped from Ciudad de Mexico inside an insulated cooler containing gel ice packs, at a temperature of 4 °C or lower. The samples were packaged in 50 mL centrifuge tubes and stored under refrigeration to later be processed.

The pH, acidity, protein content, fat content and total solid content of the whole and skim milk samples were analyzed. Briefly, the pH was evaluated with a potentiometer (Orion Star A211, Thermo Scientific, Waltham, MA, USA), and its determination was done in triplicate. Titratable acidity, fat content and total solid content were determined according to the Official Mexican Norm, NOM-155-SCFI-2012 [15]. Titratable acidity was determined by adding 2 mL of phenolphthalein to 20 mL of a milk sample; then the mixture was titrated with NaOH 0.1 N until a change in color occurred. Acidity values were expressed in g/L of lactic acid equivalents. The protein content was determined by the micro-Kjeldahl method according to NOM-155-SCFI-2012 [23], by placing 5 mL of each sample into a Kjeldahl flask and adding 12 g of potassium sulfate, 1 g of copper sulfate pentahydrate and 20 mL of sulfuric acid. The flask was then placed on a rack for digestion for 2–3 h. At the end of the digestion, the flask was allowed to cool, and 85 mL of distilled water was added. Subsequently, distillation was carried out by neutralizing the acid with 50% NaOH. Gaseous nitrogen was recovered in a flask containing 50 mL of a 4% boric acid solution with the Wesslob indicator. It was distilled for about 5 min. The distillate was titrated with 0.02 N HCl. The nitrogen present in the sample was expressed as a percentage of milliequivalents of nitrogen, and the percentage of protein was obtained by multiplying the percentage of nitrogen obtained, expressed in weight/weight, by the factor 6.38. To convert the percentage of protein to g/L, it was multiplied by the density of the milk. The milk fat content was determined by placing 10 mL of H_2_SO_4_, 11 mL of milk at 20 °C, and 1 mL of isoamyl alcohol in a butyrometer, which was then tightly sealed and vigorously shaken. The butyrometer was then placed in a centrifuge for 5 min at 515× *g*, then moved to a water bath at 65 °C for 5 to 10 min. The fat content was read on the butyrometer scale. This evaluation was carried out in duplicate. The total solid content of milk consists of lactose, proteins and minerals. First, 5 g of each milk sample was placed in a tared crucible, which was placed in a water bath until the sample was visibly dry; next, the crucible was placed in a drying oven at 102 °C until constant weight was achieved. Finally, the total solids were calculated by the moisture percentage.

### 3.2. High-Pressure Processing

Raw milk samples of 25 mL were packaged, vacuum-sealed in polyethylene bags in duplicate and placed inside an Avure 2L (Columbus, OH, USA) HPP equipment; distilled water (25 °C) was utilized as the pressure-transmitting medium. The treatment conditions were: 400 MPa during the Come-Up Time (CUT), 2, 5, 10 and 15 min, 500 MPa during the CUT, for 2, 5, 8 and 10 min and 600 MPa during the CUT, for 2, 5, 8 and 10 min. The CUT values were 1.37, 1.62 and 1.68 min for 400, 500 and 600 MPa, respectively. The aforementioned treatment conditions were determined in a previous study, in which the optimal pasteurization parameters were established [24]. Twenty-eight different high pressure-treated milk samples were analyzed in this study, as displayed in Table 3 alongside the average temperature values of the water during each treatment.

### 3.3. Scanning Electron Microscopy (SEM)

SEM was used to visualize microscopical changes in the HPP protein micelles of the treated milk samples. Adapting the methodology followed by Cadesky et al. [11] and utilizing the information regarding the preparation of liquid biological samples by Murtey and Ramasamy [25], the following preparation procedure was used. First, the milk samples were defatted by subjecting them to a double centrifugation process at 42,000× *g* for 1 h at 15 °C and removing the milk fat in the supernatant; the process was performed twice in the same conditions for 15 min. Then, the supernatant was collected to be fixed onto aluminum holds pins. For the fixation, 0.1% poly-L-lysine was first applied to completely cover the surface of the aluminum pin for 60 min and then rinsed with distilled water. Then, 100 µL of the defatted milk supernatant was placed on the pin for 60 min, followed by rinsing with 100% ethanol. Glutaraldehyde at a concentration of 4% was then added to the pin surface for 60 min, followed once again by rinsing with 100% ethanol. Lastly, osmium tetroxide at 1% was added for an additional 60 min and then washed using 100% ethanol. Then, a dehydration process was carried out by applying ethanol at ascending concentrations (50, 70 and 90%) to the pin surface for 10 min each time, rinsing with 100% ethanol between each application. Finally, 100% ethanol was applied to the pin surface three times for 5 min each time, rinsing with 100% ethanol between each application and allowing the pin to completely dry, before storing on a desiccator. The aluminum pins with the fixed samples were coated with gold particles (Quorum Q150R ES) and placed in the SEM equipment (EVO MA25 ZEISS, Germany) for observation. The measurement of the size of the micelles was carried out using the software of the SEM equipment. For each sample, three micrographs were obtained. The mean and standard deviation were obtained by observing each sample in different fields. The distribution analysis consisted in quantifying the size distribution frequency in five different ranges, i.e., less than 100 nm, from 100 to 200 nm, from 200 to 300 nm, from 300 to 400 nm and greater than 400 nm, for each different sample.

### 3.4. Statistical Analysis

A one-way ANOVA with a 95% confidence level and a particle size analysis were conducted using the software package Minitab version 18.

## 4. Conclusions

Microscopical analysis demonstrated that casein micelles are affected by high-pressure treatments. High-pressure-treated casein micelle presented three different phenomena: fragmentation, agglomeration and alteration to their shapes. In general, the extent of these effects increased as the pressure and time treatments increased. Particle size distribution analysis revealed that as pressure intensity and treatment time increased, the three phenomena became more evident. Additionally, milk pretreatments, such as homogenization and skimming, had no significant effect on casein micelle size; however, the presence of fat had a baroprotective effect on the casein micelles in whole milk samples. Further thorough analyses are recommended regarding the mean diameter size and the particle size distribution of casein micelles. To establish how these changes in size and composition affect the physicochemical and sensorial properties and the stability and shelf life of treated milk and to determine the specific physical and chemical interaction between the different components of casein micelles during HPP and the specific mechanisms leading to the three described phenomena more research is necessary.

## Figures and Tables

**Table 1:** Physicochemical parameters of the whole and skim milk samples.

Parameter	Whole Milk	Skim Milk
**pH**	6.65 ± 0.05	6.70 ± 0.02
**Acidity (g/L, (lactic acid equivalents)**	1.62 ± 0.00	1.64 ± 0.03
**Protein (g/L)**	29.30 ± 0.90	31.30 ± 1.10
**Fat (g/L)**	3.50 ± 0.00	1.00 ± 0.00
**Total Solids (g/L)**	11.35 ± 0.03	11.31 ± 0.11

**Table 2:** Diameter (nm) of the casein micelles obtained from the SEM micrographs of the whole and skimmed milk samples.

Treatments	Whole Milk	Skim Milk
	Mean Diameter (nm)	
**Homogenized and untreated**	391 ± 112 ^a^	370 ± 266 ^bc^
**Untreated**	370 ± 175 ^ab^	ND
**400 MPa, CUT**	319 ± 82 ^abcd^	233 ± 146 ^c^
**400 MPa, 2 min**	169 ± 9 ^cd^	408 ± 204 ^abc^
**400 MPa, 5 min**	159 ± 109 ^cd^	263 ± 129 ^c^
**400 MPa, 10 min**	178 ± 142 ^bcd^	281 ± 129 ^c^
**400 MPa, 15 min**	208 ± 114 ^bcd^	588 ± 254 ^a^
**500 MPa, CUT**	273 ± 121 ^abcd^	322 ± 130 ^c^
**500 MPa, 2 min**	190 ± 97 ^bcd^	448 ± 190 ^abc^
**500 MPa, 5 min**	198 ± 159 ^bcd^	587 ± 146 ^ab^
**500 MPa, 8 min**	ND	625 ± 170 ^ab^
**500 MPa, 10 min**	284 ± 165 ^abc^	313 ± 118 ^c^
**600 MPa, CUT**	237 ± 130 ^abcd^	238 ± 117 ^c^
**600 MPa, 2 min**	135 ± 72 ^d^	252 ± 132 ^c^
**600 MPa, 5 min**	166 ± 123 ^cd^	374 ± 182 ^abc^
**600 MPa, 8 min**	ND	268 ± 137 ^c^
**600 MPa, 10 min**	239 ± 148 ^abcd^	236 ± 147 ^c^

Values in the same column for each sample type with different letters differ significantly (*p* < 0.05). CUT stands for Come-Up Time. ND stands for no data.

**Table 3:** Average temperature (*T*) during the high-hydrostatic-pressure treatment of milk. Initial water temperature, 21.6 ± 1.6 °C.

HPP Treatment		*T* (°C)
P (MPa)	t (min)	
400	CUT	26.2 ± 3.6
	2	34.1 ± 11.1
	5	30.6 ± 4.2
	10	29.3 ± 4.7
	15	28.6 ± 3.8
500	CUT	23.9 ± 5.5
	2	31.7 ± 6.6
	5	31.9 ± 6.6
	8 *	30.2 ± 6.4
	10	29.4 ± 5.8
600	CUT	25.3 ± 10.8
	2	33.3 ± 7.5
	5	33.3 ± 7.5
	8 *	31.5 ± 7.5
	10	31.1 ± 7.3

* All treatments were applied to both milk types, except for those marked with ana asterisk, which were applied exclusively to skim milk.

## Data Availability

Not applicable.

## Sample Availability

Samples of the compounds are not available from the authors.

## References

[1] Stratakos A.C., Inguglia E.S., Linton M., Tollerton J., Murphy L., Corcionivoschi N., Koidis A., Tiwari B.K. (2019). Effect of high pressure processing on the safety, shelf life and quality of raw milk. Innov. Food Sci. Emerg. Technol..

[2] Roobab U., Shabbir M.A., Khan A.W., Arshad R.N., Bekhit A.E., Zeng X., Inam-Ur-Raheem M., Aadil R.M. (2021). High-pressure treatments for better quality clean-label juices and beverages: Overview and advances. LWT.

[3] Nabi B.G., Mukhtar K., Arshad R.N., Radicetti E., Tedeschi P., Shahbaz M.U., Walayat N., Nawaz A., Inam-Ur-Raheem M., Aadil R.M. (2021). High-Pressure Processing for Sustainable Food Supply. Sustainability.

[4] Liu G., Carøe C., Qin Z., Munk D.M.E., Crafack M., Petersen M.A., Ahrné L. (2020). Comparative study on quality of whole milk processed by high hydrostatic pressure or thermal pasteurization treatment. LWT.

[5] Serna-Hernandez S.O., Escobedo-Avellaneda Z., García-García R., de Rostro-Alanis M.J., Welti-Chanes J. (2021). High hydrostatic pressure induced changes in the physicochemical and functional properties of milk and dairy products: A review. Foods.

[6] Özer B., Yaman H. (2014). Milk and Milk Products: Microbiology of Liquid Milk. Encycl. Food Microbiol. Second Ed..

[7] Chavan R.S., Sehrawat R., Mishra V., Bhatt S. (2015). Milk: Processing of Milk.

[8] Liepa M., Zagorska J., Galoburda R. (2016). High-pressure processing as novel technology in dairy industry: A review. Res. Rural Dev..

[9] Dalgleish D.G., Corredig M. (2012). The Structure of the Casein Micelle of Milk and Its Changes During Processing. Annu. Rev. Food Sci. Technol..

[10] Bhat M.Y., Dar T.A., Singh L.R. (2016). Casein Proteins: Structural and Functional Aspects. Milk Proteins—From Structure to Biological Properties and Health Aspects.

[11] Cadesky L., Walkling-Ribeiro M., Kriner K.T., Karwe M.V., Moraru C.I. (2017). Structural changes induced by high-pressure processing in micellar casein and milk protein concentrates. J. Dairy Sci..

[12] Trejo R., Dokland T., Jurat-Fuentes J., Harte F. (2011). Cryo-transmission electron tomography of native casein micelles from bovine milk. J. Dairy Sci..

[13] Ogura T., Okda T. (2017). Nanoscale observation of the natural structure of milk-fat globules and casein micelles in the liquid condition using a scanning electron assisted dielectric microscopy. Biochem. Biophys. Res. Commun..

[14] Hemar Y., Xu C., Wu S., Ashokkumar M. (2020). Size reduction of “reformed casein micelles” by high-power ultrasound and high hydrostatic pressure. Ultrason. Sonochem..

[15] DOF NORMA Oficial Mexicana NOM-155-SCFI-2012, Leche-Denominaciones, Especificaciones Fisicoquímicas, Información Comercial y Métodos de Prueba. https://www.dof.gob.mx/normasOficiales/4692/seeco/seeco.htm.

[16] Yang S., Liu G., Munk D.M.E., Qin Z., Petersen M.A., Cardoso D.R., Otte J., Ahrné L. (2020). Cycled high hydrostatic pressure processing of whole and skimmed milk: Effects on physicochemical properties. Innov. Food Sci. Emerg. Technol..

[17] Naik L., Sharma R., Rajput Y., Manju G. (2013). Application of High Pressure Processing Technology for Dairy Food Preservation—Future Perspective: A Review. J. Anim. Prod. Adv..

[18] Goyal A., Sharma V., Upadhyay N., Sihag M., Kaushik R. (2013). High Pressure Processing and Its Impact on Milk Proteins: A Review. Res. Rev. J. Dairy Sci. Technol..

[19] Huppertz T., Fox P.F., de Kruif K.G., Kelly A.L. (2006). High pressure-induced changes in bovine milk proteins: A review. Biochim. Biophys. Acta Proteins Proteom..

[20] Otto T., Sicinski P. (2017). Cell cycle proteins as promising targets in cancer therapy. Nat. Rev. Cancer.

[21] Munir M., Nadeem M., Qureshi T.M., Leong T.S.H., Gamlath C.J., Martin G.J.O., Ashokkumar M. (2019). Effects of high pressure, microwave and ultrasound processing on proteins and enzyme activity in dairy systems—A review. Innov. Food Sci. Emerg. Technol..

[22] Huppertz T., Fox P.F., Kelly A.L. (2004). High pressure treatment of bovine milk: Effects on casein micelles and whey proteins. J. Dairy Res..

[23] DOF NORMA Oficial Mexicana NOM-243-SSA1-2010, Productos y Servicios. Leche, Fórmula Láctea, Producto Lácteo Combinado y Derivados Lácteos. Disposiciones y Especificaciones Sanitarias. Métodos de Prueba. https://dof.gob.mx/normasOficiales/4156/salud2a/salud2a.htm.

[24] Serna-Hernandez S.O. (2021). Effects of High Hydrostatic Pressure in the Microbiological, Microscopical and Physicochemical Properties of Milk.

[25] Das Murtey M., Ramasamy P., Janecek M., Kral R. (2016). Sample Preparations for Scanning Electron Microscopy—Life Sciences. Modern Electron Microscopy in Physical and Life Sciences.

